# The ascending arousal system promotes optimal performance through mesoscale network integration in a visuospatial attentional task

**DOI:** 10.1162/netn_a_00205

**Published:** 2021-11-30

**Authors:** Gabriel Wainstein, Daniel Rojas-Líbano, Vicente Medel, Dag Alnæs, Knut K. Kolskår, Tor Endestad, Bruno Laeng, Tomas Ossandon, Nicolás Crossley, Elie Matar, James M. Shine

**Affiliations:** Brain and Mind Centre, University of Sydney, Sydney, NSW, Australia; Centro de Neurociencia Humana y Neuropsicología, Facultad de Psicología, Universidad Diego Portales, Santiago, Chile; Brain and Mind Centre, University of Sydney, Sydney, NSW, Australia; Department of Psychiatry, School of Medicine, Pontificia Universidad Católica de Chile, Santiago, Chile; NORMENT, Division of Mental Health and Addiction, University of Oslo, and Oslo University Hospital, Oslo, Norway; Bjørnnes College, Oslo, Norway; NORMENT, Division of Mental Health and Addiction, University of Oslo, and Oslo University Hospital, Oslo, Norway; Department of Psychology, University of Oslo, Oslo, Norway; Sunnaas Rehabilitation Hospital HT, Nesodden, Norway; Department of Psychology, University of Oslo, Oslo, Norway; RITMO Centre for Interdisciplinary Studies in Rhythm, Time, and Motion, University of Oslo, Oslo, Norway; Helgelandssykehuset Mosjøen, Helse Nord, Norway; Department of Psychology, University of Oslo, Oslo, Norway; RITMO Centre for Interdisciplinary Studies in Rhythm, Time, and Motion, University of Oslo, Oslo, Norway; Department of Psychiatry, School of Medicine, Pontificia Universidad Católica de Chile, Santiago, Chile; Institute for Biological and Medical Engineering, Schools of Engineering, Medicine and Biological Sciences, Pontificia Universidad Católica de Chile, Santiago, Chile; Department of Psychiatry, School of Medicine, Pontificia Universidad Católica de Chile, Santiago, Chile; Brain and Mind Centre, University of Sydney, Sydney, NSW, Australia; Brain and Mind Centre, University of Sydney, Sydney, NSW, Australia; Centre for Complexity, University of Sydney, Sydney, NSW, Australia

**Keywords:** Pupil diameter, Locus coeruleus, Network integration, Noradrenergic system, Neuromodulation, Attention, Mental effort, fMRI

## Abstract

Previous research has shown that the autonomic nervous system provides essential constraints over ongoing cognitive function. However, there is currently a relative lack of direct empirical evidence for how this interaction manifests in the brain at the macroscale level. Here, we examine the role of ascending arousal and attentional load on large-scale network dynamics by combining pupillometry, functional MRI, and graph theoretical analysis to analyze data from a visual motion-tracking task with a parametric load manipulation. We found that attentional load effects were observable in measures of pupil diameter and in a set of brain regions that parametrically modulated their BOLD activity and mesoscale network-level integration. In addition, the regional patterns of network reconfiguration were correlated with the spatial distribution of the α2a adrenergic receptor. Our results further solidify the relationship between ascending noradrenergic activity, large-scale network integration, and cognitive task performance.

## Author Summary

In our daily lives, it is usual to encounter highly demanding cognitive tasks. They have been traditionally regarded as challenges that are solved mainly through cerebral activity, specifically via information-processing steps carried by neurons in the cerebral cortex. Activity in cortical networks thus constitutes a key factor for improving our understanding of cognitive processes. However, recent evidence has shown that evolutionary older players in the central nervous system, such as brain stem’s ascending modulatory systems, might play an equally important role in diverse cognitive mechanisms. Our article examines the role of the ascending arousal system on large-scale network dynamics by combining pupillometry, functional MRI, and graph theoretical analysis.

## INTRODUCTION

Cognitive processes emerge from the dynamic interplay between diverse mesoscopic brain systems (Shine, 2021; Shine et al., 2016). Thus, the neural activity supporting cognition does not exist in a vacuum, but instead is deeply embedded within the ongoing dynamics of the physiological networks of the body (Varela et al., 2001). In particular, the neural processes underlying cognition are shaped and constrained by the ascending arousal system, whose activity acts to facilitate the integration between internal states and external contingencies (Parvizi & Damasio, 2001). Timely and selective interactions between the ascending arousal system and the network-level configuration of the brain are thus likely to represent crucial constraints on cognitive and attentional processes. Yet, despite these links, we currently have a relatively poor understanding of how the ascending arousal system helps the brain as a whole to functionally reconfigure during cognitive processes, such as attention, in order to facilitate effective cognitive performance.

Recent evidence has linked higher order cognitive functions in the brain to the intersection between whole-brain functional network architecture and the autonomic arousal system (Alnæs et al., 2015; Alnæs et al., 2014; Munn et al., 2021; Shine et al., 2016; Shine, Hearne, et al., 2019). Central to these relationships is the unique neuroanatomy of the ascending noradrenergic system. For instance, the pontine locus coeruleus, which is a major hub of the ascending arousal system, sends widespread projections to the rest of the brain (Samuels & Szabadi, 2008). Upon contact, adrenergic axons release noradrenaline, which acts as a ligand on three types of post- and presynaptic adrenergic receptors (i.e., α1, α2, and β). The functional effects of each of these receptors depend on their differential sensitivities to noradrenaline (affinities for the ligand differ across receptors: α2 > α1 > β) and intracellular cascades, as well as their neuronal and regional distributions (Aston-Jones & Waterhouse, 2016; Bouret & Sara, 2005; Robbins & Arnsten, 2009; Samuels & Szabadi, 2008; Sara, 2009; Shine, 2019). By modulating the excitability of targeted regions, the locus coeruleus can effectively coordinate neural dynamics across large portions of the cerebral cortex (Shine et al., 2021; X. J. Wang, 2020). However, it is challenging to noninvasively track the engagement of the locus coeruleus during whole-brain neuroimaging and cognitive task performance.

Fortunately, it has been widely shown that the pupil diameter directly responds to changes in the activity of the locus coeruleus, and thus serves as an indirect, noninvasive measure of the noradrenergic system (Aston-Jones & Cohen, 2005; S. Joshi et al., 2016). Specifically, pupil diameter has been shown to indirectly monitor the neuromodulatory influences of the ascending arousal system on a variety of different brain regions (Alnæs et al., 2014; Liu et al., 2017; Sara, 2009; van den Brink et al., 2016). Moreover, noradrenergic-mediated dilations in pupil diameter have been shown to effectively track the allocation of attentional resources (Gilzenrat et al., 2010; Kahneman & Beatty, 1966; Wainstein et al., 2017), in addition to both physical and mentally effortful processes (Mulder, 2012; Varazzani et al., 2015). Fast, phasic changes in pupil diameter have also been shown to directly relate to changes in the activity of the locus coeruleus (S. Joshi et al., 2016; Murphy et al., 2016; Reimer et al., 2014). While there is some evidence that pupil diameter covaries with other subcortical systems (S. Joshi & Gold, 2020), such as the cholinergic and serotoninergic system (Cazettes et al., 2020), the physiological mechanism for these effects is more opaque, and there is also clear causal evidence linking stimulation of the locus coeruleus to dilation of the pupil (Liu et al., 2017; Zerbi et al., 2019). Despite these insights, several questions remain unanswered regarding how these processes are related to the complex architecture of the brain (Shenhav et al., 2017). For instance, the processes by which the ascending arousal system modulates the functional dynamics of brain networks to facilitate attention, decision-making, and optimal behavioral performance have only begun to be explored (de Gee et al., 2017; Shine, Breakspear, et al., 2019; Shine et al., 2018; Zerbi et al., 2019).

**Figure 1.**
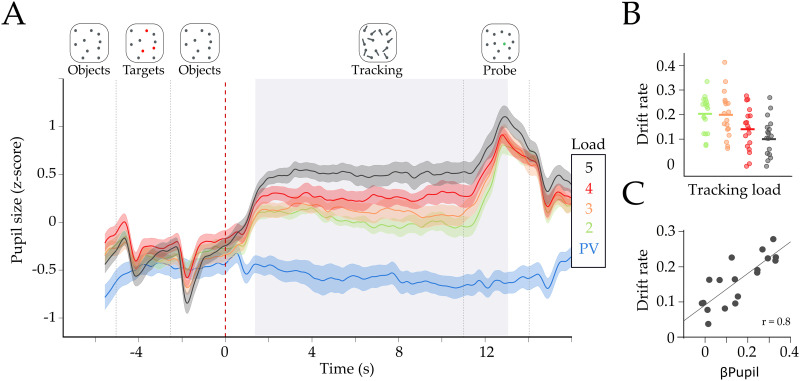
**Effect of task difficulty on pupil diameter.** (A) Group average (z-score) pupil diameter time series for each load condition. Colors represent passive viewing (PV) in blue, and Loads 2 to 5 in green, orange, red, and black, respectively. The shaded area represents the standard error of the mean. We observed an average increase in pupil diameter, during tracking, with each load condition. The light gray area represents time points with significant parametric effect (β_pupil_ > 0; FDR corrected at *p* < 0.01). Dotted lines represent the onset of each trial event (shown in the top part of the figure). The red dotted line (time = 0) is the tracking onset period when the dots began to move. (B) Drift rate in each load condition. Each dot is the drift rate for each subject and load (mean β_Drift_ = −0.03, *t*_(17)_ = −7.43, *p* = 9.7 × 10^−7^). (C) Pearson correlation between the pupil parametric effect of load (β_pupil_) with the average drift rate across subjects (r_drift_ = 0.8, *p* = 1.0 × 10^−4^). The x-axis is the mean beta estimate of the pupillary load effect of the significative time window (β_pupil_), and the y-axis represents the mean drift rate across loads.

To examine these relationships in more detail, participants performed a motion-tracking task (top panel of Figure 1A) involving four levels of increasing attentional load, which was modulated by manipulating the number of items required to covertly attend to over an 11-s tracking period. Specifically, subjects were instructed to covertly track the movement of several preidentified targets (two to five) in a field of nontarget stimuli (10 in total, including targets; see Figure 1). To investigate the network topological signatures of performing this task, we collected concurrent BOLD fMRI and pupillometry data. We hypothesized that, if increasing mental effort led to the reconfiguration of large-scale network architecture via the ascending arousal system, then the number of items required to be tracked over time (i.e., the attentional load) should relate to (a) increased pupil diameter; (b) heightened BOLD activity within attentional networks; and (c) augmented topological integration. Also, we predicted that individual differences in pupil diameter should track individual differences in effective attentional performance and decision processes (de Gee et al., 2017; de Gee et al., 2014; Donner et al., 2000). Finally, we tested whether the regional patterns of network configuration were predicted by the distribution of a predefined adrenergic receptor density atlas (Fornito et al., 2019; Richiardi et al., 2015; Shine, Breakspear, et al., 2019; Zerbi et al., 2019). Our results confirm these predictions, and hence provide a mechanistic link between network topology, ascending noradrenergic arousal, and attentional load.

## RESULTS

### The Relationship Between Sympathetic Tone and Attentional Processing

Consistent with previous work (Alnæs et al., 2014), our two-level analysis—linear regression within each subject, and a two-tailed *t* test between subjects—found that task performance (i.e., correct responses) decreased with attentional load (mean β_Acc_ = −6.66; *t*_(17)_ = −5.19, *p* = 7.2 × 10^−5^; Supplementary Figure S1B) while the reaction time (RT) increased with attentional load (mean β_RT_ = 0.06, *t*_(17)_ = 5.10, *p* = 8.8 × 10^−5^). We expanded on this result by translating performance into EZ-diffusion model parameters. Roughly, this approach uses the accuracy and reaction time distribution to estimate three latent parameters (de Gee et al., 2014): drift rate, a marker of the accumulation of decision evidence (Equation 1); boundary criteria, the amount of evidence required to make a decision (Equation 2); and non-decision time, the epoch spent processing the tasks perceptually (Equation 3). The advantages of using this model are twofold: first, there are well-known links between the parameters to decision-making processes (Ratcliff et al., 2016; Ratcliff et al., 2015), pupil diameter (Murphy et al., 2016; Murphy et al., 2014) and network reconfiguration (Shine et al., 2016); second, drift rate accounts for the accuracy–reaction time trade-off, as it takes into consideration both accuracy and the variability in reaction time into its calculation. In this way, our approach offers a better approximation of the ongoing computational processing during the task than does accuracy and RT (Ratcliff et al., 2015; Wagenmakers et al., 2007). Using this approach, we observed a decrease in both the boundary criteria (β_Bound_ = −0.01, *t*_(17)_ = −2.70, *p* = 0.015) and the drift rate (mean β_Drift_ = −0.03, *t*_(17)_ = −7.43, *p* = 9.7 × 10^−7^; Figure 1B), and an increase in the non-decision time (mean β_nd_ = 0.07, *t*_(17)_ = 5.32, *p* = 5.5 × 10^−5^) with increasing attentional load.

By calculating the linear effect of load on pupil size across a moving average window of 160 ms (see Methods), we observed a main effect of increased pupil diameter across both the tracking and the probe epochs (β_pupil_ > 0, *p*_FDR_ < 0.01; light gray area in Figure 1A depicts significant epochs of time during the task; and in Supplementary Figure S1A shows the group average β_pupil_ time series). We also observed a positive correlation between mean β_pupil_ during the significant period (for simplicity we will refer to this value as βpupil) to the mean drift rate, mean boundary criteria, and accuracy across all loads (Pearson’s *r*_drift_ = 0.8, *p* = 1.0 × 10^−4^; Figure 1C; r_acc_ = 0.68, *p* = 1.5 × 10^−3^, Supplementary Figure S1C; *r*_Bound_ = 0.71, *p* = 9 × 10^−4^). The same relationships were not observed with non-decision time (Pearson’s *r*_nd_ = −0.31, *p* = 0.19). Additionally, we analyzed whether this effect was present both within and between subjects in a trial-by-trial manner. To this end, we created a logistic linear mixed model (Equation 6) to test whether pupil diameter was a predictor of performance (i.e., correct or incorrect response), as we would expect that incorrect responses should relate to decreased pupil diameter in difficult trials. We used the average pupil diameter within each trial of Load 4 and 5 (to account for the ceiling effect of Load 2 and 3) as regressors and subject as a grouping variable. We found a statistically significant fixed effect of pupil diameter on performance within each trial (β = 0.0127 ± 5 × 10^−4^; *t*_(286)_ = 2.48; *p* = 0.013). Furthermore, we analyzed the random-effect coefficients, which are the dispersion of the regressor across the grouping variable from the fixed regressor (in this case there is one value per subject), to assess the role of average across task performance. We found that the random effect covaried with the average performance and drift rate of each subject (Accuracy: Pearson’s *r* = 0.73, *p* = 8 × 10^−5^; Drift: Pearson’s *r* = 0.73, *p* = 5 × 10^−5^), suggesting that trial-by-trial pupil diameter was a better predictor of performance (i.e., correct or incorrect) on subjects with higher average performance in comparison to subjects with lower performance across the task. In conclusion, these results suggest that attentional load manipulation and pupil dilation covaried with performance on this attentionally demanding task both within and between subjects.

### Network Integration Increases as a Function of Attentional Load

**Figure 2.**
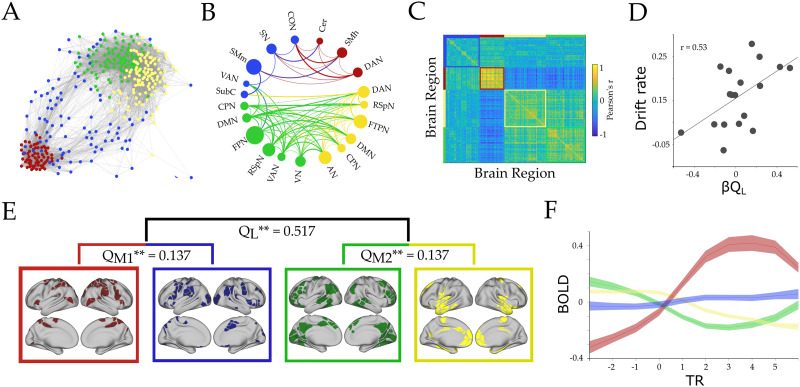
**Hierarchical functional topology analysis of the brain during tracking across all loads.** We observed two large-scale modules and two mesoscale modules within each larger module (Module 1 [M1, red/blue] and Module 2 [M2, green/yellow], respectively): M1 corresponded to predominantly attentional and somatomotor network, and M2 to frontoparietal network (FPN) and default mode network (DMN), among others (B and E). (A) Forced directed plot representation of the average cluster across subjects. Edges stronger than 0.3 are shown. Each color represents a unique submodule. (B) A circle plot representing the resting-state regions that were included within each submodule, with networks with >30% of regions in each submodule shown in the plot. The diameter of the circles corresponds to the percentage of network regions that participated in that cluster. Connection width relates to average positive connection strength (functional connectivity); however, only connections with *r* > 0.1 are shown. (C) Connectivity matrix (Pearson’s *r*) between all pairs of regions ordered by module assignments—note the strong anticorrelation between the red and green/yellow submodules. (D) Correlation between parametric load effect on large-scale modularity (β_Q_ value) and drift rate (Pearson’s *r* = 0.53; *p* = 0.022). (E) Hierarchical analysis representation: *Q*_L_, *Q*_M1_, and *Q*_M2_ represent the modularity value for each level (*Q*_L_ large-scale, and *Q*_M1–M2_ mesoscale level), and ** represents the probability of finding this value when running a null model (*p* = 0 for all three modularity values). The brain maps correspond to the cortical regions associated with each submodule. (F) BOLD mean effect for each subcluster. Each line represents the group average, and shaded areas are the standard error of the mean. X-axis is repetition time (TR) centered around tracking onset (TR = 0). DAN, dorsal attention; VN, visual; FPN, frontoparietal; SN, salience; CO, cingulo-opercular; VAN, ventral attention; SMm, somatomotor mouth; SMh, somatomotor hand; RSpN, retrosplenial; FTP, frontotemporal; DMN, default mode; AN, auditory; CPN, cinguloparietal; SubC, subcortex; Cer, cerebellar.

Based on previous studies, we hypothesized that an increase in attentional load should recruit a distributed functional network architecture (Alnæs et al., 2014), heightening network integration (Shine, 2019; Shine et al., 2016; Shine, Breakspear, et al., 2019). To test this hypothesis, we implemented a hierarchical topological network analysis (Bassett et al., 2010; Meunier et al., 2010; Meunier et al., 2009) on the average time-resolved functional connectivity matrix calculated across the tracking period of the task. Our analysis identified a subnetwork of tightly interconnected regions that were part of attentional, somatomotor, and cerebellar network (red in Figure 2) that increased its BOLD activity after the tracking onset (Figure 2F). The tightly integrated regions were diversely connected to a separate frontoparietal submodule (blue in Figure 2) that was less active during the trial. Two remaining submodules (yellow and green in Figure 2) showed a negative BOLD response during the tracking period and were part of a diverse set of networks. Interestingly, 81% of the frontoparietal network (FPN) and all the default mode network (DMN) were found to be within this less active group (see Supplementary Table S2 for the complete list of regions and submodule assignments).

Contrary to expectations, we did not observe significant parametric topological change (i.e., modularity, Q) at the macroscopic level as a function of attentional load (*p* > 0.05 for all TRs, Supplementary Figure S2A). However, when analyzing the correlation between modularity and performance measures (i.e., accuracy, drift rate, and pupil diameter), we observed that an increase in the large-scale modularity load effect (i.e., higher modularity with load, β_*Q*L_) positively correlated with higher mean drift rate (Pearson’s *r* = 0.53; *p* = 0.022; Figure 2D), mean accuracy (Pearson’s *r* = 0.61; *p* = 0.007; Supplementary Figure S3A), but was independent from βpupil (Pearson’s *r* = 0.43; *p* = 0.073). These results suggested that the system reconfigured during tracking towards increasing modularity, which in turn affected the efficient encoding of the ongoing task during tracking and hence, the decision-making process during the task probe.

**Figure 3.**
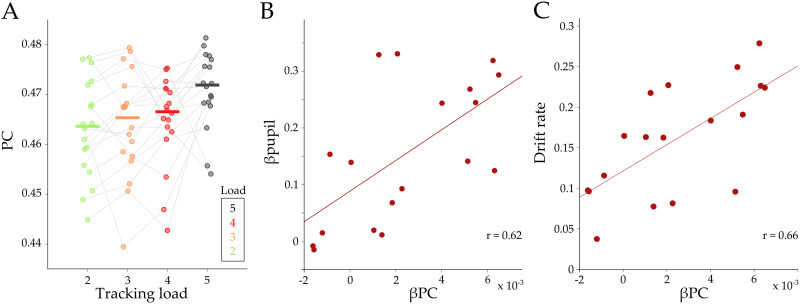
**Relationships between load effect on participation, pupil load effect, and drift rate.** (A) Average participation coefficient (PC) for each load, for the red module, during tracking. Each color represents the corresponding tracking load (from 2 to 5). Gray lines correspond to each subject. (B–C) A regression parameter (βPC) was calculated for each subject and then correlated to βpupil (B; *r* = 0.62; *p* = 0.006) and drift rate (C; *r* = 0.66; *p* = 2.4 × 10^−3^). Each circle corresponds to the mean value per subject.

Upon closer inspection of the data (Figure 2C), we observed a substantial number of nodes that were playing an integrative role during task performance, albeit at a finer resolution than the initial analysis suggested. We performed the modularity assignment within each large-scale module. The hierarchical analysis resulted in two pairs of submodules at the mesoscale level with a significant modularity (compared with 100 random graphs with preserved signed degree distribution; Q_M1_ = 0.137, *p* = 0; Q_M2_ = 0.137, *p* = 0; Figure 2E). Specifically, the red submodule was found to selectively increase its participation coefficient (PC) at the mesoscale level (i.e., by increasing the connection weights to the blue submodule in comparison with intramodular connections; Equation 5) as a function of increasing attentional load (β_PC_ = 2.4 × 10^−3^, *t*_(17)_ = 3.57; *p* = 0.002; Figure 3A). Additionally, the extent of integration in the red submodule was positively correlated across subjects with βpupil (Pearson’s *r* = 0.62, *p* = 0.006; Figure 3B), drift rate (Pearson’s *r* = 0.66, *p* = 0.002; Figure 3C), and accuracy (Pearson’s *r* = 0.57, *p* = 0.012, Supplementary Figure S3B). Importantly, these relationships were found to be specific to the red submodule (blue: Pearson’s *r* = −0.02, *p* = 0.936; yellow: Pearson’s *r* = −0.011, *p* = 0.965; green: Pearson’s *r* = −0.12, *p* = 0.617).

Based on these results, we implemented a linear mixed model (Equation 7), using the subjects’ average pupil response within each load as a regressor and the average participation of the red submodule as the dependent variable, with grouping by subject. Using this approach, we observed a significant fixed effect of pupil diameter on PC (β = 7.6 × 10^−3^ ± 3 × 10^−3^, *t*_(70)_ = 2.60, *p* = 0.011). Furthermore, the random-effect coefficients (i.e., the between-subject variation of the regressor value) correlated positively with accuracy (Pearson’s *r* = 0.47, *p* = 0.048) and drift rate (Pearson’s *r* = 0.62, *p* = 0.005), suggesting that subjects with a strong relationship between red module integration and pupil diameter have better behavioral outcomes. We then correlated the red βPC to the load effect on large-scale modularity (βQ_L_, Figure 2D) and observed a significant positive correlation (Pearson’s *r* = 0.59, *p* = 0.009). Finally, given that both topological parameters were correlated with drift rate and also with each other, we performed a partial correlation between drift rate and βPC controlling by βQ_L_ (*r* = 0.51, *p* = 0.034), and the partial correlation between drift rate and βQ_L_ controlling by βPC (*r* = 0.36, *p* = 0.145). This suggests that drift rate is correlated with the mesoscale integration of the red submodule, but less so with increases in large-scale modularity. Thus, although the macroscale network did not demonstrate increased integration per se, the relative amount of mesoscale integration within the red community was associated with increased performance (i.e., drift rate) and sympathetic arousal (i.e., pupil diameter), both between and within subjects. In this way, these results provide a direct relationship between the effect of attention load on pupillometry, drift rate, and a trade-off between large-scale segregation and mesoscale network integration.

### Network Mesoscale Integration and Adrenergic Receptor Density

Given the relationship between mental effort, noradrenergic tone, and pupil dilation (Alnæs et al., 2014; S. Joshi et al., 2016; McGinley et al., 2015; Reimer et al., 2014; Varazzani et al., 2015), the results of our analyses strongly suggested that the adrenergic system is involved in the mesoscale network reconfiguration observed during attentional tracking. The locus coeruleus can impact the cortical system in multiple ways, both through direct release of noradrenaline onto cortical neurons, and through the modulation of subcortical regions (such as the thalamic nuclei) with concurrent impact on the cortical dynamic. Importantly, in either case, the modulation is dependent on the noradrenergic receptors subtypes, which have different sensitivities to noradrenaline (Robbins & Arnsten, 2009; M. Wang et al., 2007) and variable expression in the cerebral cortex (Santana & Artigas, 2017; Zilles & Palomero-Gallagher, 2017), and also belong to distinct classes (i.e., α1, α2, and β receptors). In particular, the α2a has been previously associated with working memory, adaptive gain, and effective attention (Arnsten et al., 2012; Robbins & Arnsten, 2009; M. Wang et al., 2007). To gain a deeper insight into the role of α2a receptors in mesoscale integration during attentional tracking, we extracted the regional expression of the ADRA2A gene (which codes for α2a adrenoceptors) from the Allen Human Brain Atlas repository (Gryglewski et al., 2018; Hawrylycz et al., 2012), and compared the cortical regional expression of this gene with the brain activity patterns identified in our network analysis (Figure 2E).

Based on the relationships between pupil diameter (Figure 1), topological signatures (Figure 2), and task performance (Figure 3), and the known link between these variables and engagement of the noradrenergic system, we hypothesized that the different modules and submodules that we observed should have different densities of neuromodulatory receptors to account for the differential patterns across the network. To test this hypothesis, we conducted a two-tailed *t* test in each hierarchical level comparing the density of the ADRA2A expression between modules. To account for spatial autocorrelation, we generated 5,000 surrogate maps with the same spatial autocorrelation of the ADRA2A map, calculated a *t* statistic for each surrogate, and evaluated the probability of finding the observed *t* statistic against the null distribution (Burt et al., 2020; Markello & Misic, 2021). We indeed observed significant differences between modules at the mesoscale level. Specifically, we found significant differences between the blue and yellow submodules (*t*_(194)_ = 3.82, *p* = 2 × 10^−4^, *p*_SA_ = 0.02) and the differences between green and yellow submodules (*t*_(177)_ = −4.47, *p* = 1.3 × 10^−5^, *p*_SA_ = 0.004), while the other differences did not survive the spatial autocorrelation test (green-red: *t*_(152)_ = 0.47; *p* = 0.635, *p*_SA_ = 0.590; yellow-red: *t*_(156)_ = −3.02, *p* = 0.003, *p*_SA_ = 0.121; green-blue: *t*_(173)_ = −0.68, *p* = 0.496, *p*_SA_ = 0.324; red-blue: *t*_(135)_ = −1.30, *p* = 0.195, *p*_SA_ = 0.237; Supplementary Figure S5A).

**Figure 4.**
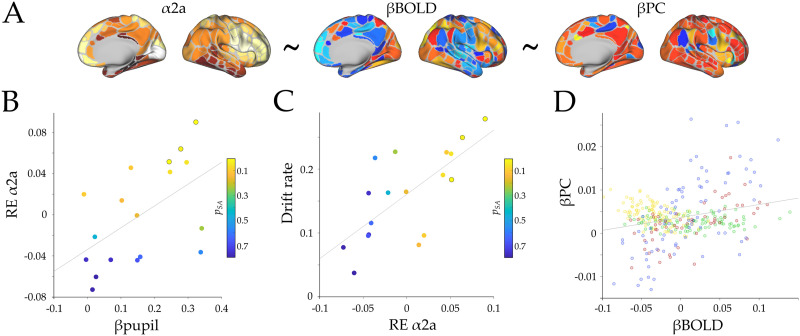
**Receptor density analysis.** (A) Spatial maps of α2a density (left), BOLD parametric effect (middle), and participation coefficient parametric effect (right). The ∼ symbol represents the linear model tested in the analysis. (B) Scatterplot depicting the relationship between β_Pupil_ and the random effect of α2a (RE α2a; *r* = 0.54, *p* = 0.02). (C) Scatterplot depicting the relationship between the random effect of α2a and drift rate (*r* = 0.70, *p* = 0.001); the colors of the dots represent the *p*_SA_ value from the linear effect of α2a on βBOLD within each subject, and the marked circles correspond to subjects with *p*_SA_ < 0.05. (D) Pearson correlation of the group average BOLD parametric effect (β_BOLD_) and participation coefficient (β_PC_; *r* = 0.26, *p* = 7 × 10^−7^). Colors represent each module assignment as in Figure 2.

The modulatory effects of noradrenaline have been argued to depend directly on ongoing glutamatergic activity in target regions (Mather et al., 2016; Shine, 2021). Moreover, it has been shown that the main source of the BOLD activity is the neurovascular response caused by pyramidal neurons containing cyclo-oxygenase-2 (Lecrux & Hamel, 2016). Importantly, this evoked response following noradrenergic activation is dependent on the ongoing activity of the pyramidal neurons (Bekar et al., 2012). Thus, the role of noradrenaline on brain dynamics and BOLD response depends critically on ongoing glutamatergic activity, which putatively represents pooled neural spiking activity (Logothetis, 2003). Given the differential task-related BOLD activity of the different submodules (i.e., Figure 2F, Supplementary Figure S4, and Figure 4A), and the observed regional variability and specificity of integration across the network, we hypothesized that network-level integration would be explained by the combined effect of ongoing BOLD activity and the distribution of the adrenergic receptor expression. Finally, we predicted that the role of the α2a receptor atlas in shaping brain activity and topology should be dependent of the subjects’ pupil diameter, such that higher βpupil should rely on a stronger relationship between network topology and α2a receptor expression.

To evaluate between these different hypotheses, we created three linear mixed models in order to better disentangle the different plausible interactions between the variables (see Methods), while still controlling for between-subject variability as a grouping variable. Additionally, to control for spatial autocorrelation, we used 5,000 surrogate maps that maintained the spatial autocorrelation of the α2a while permuting the density values. In the first model (Equation 8), we tested the hypothesis that the parametric BOLD effect (i.e., βBOLD, Supplementary Figure S4) is shaped by the distribution of α2a receptors. We found significant evidence for a positive fixed effect of α2a on βBOLD activity; however, this effect did not survive correction for spatial autocorrelation (β_α2a_ = 0.037 ± 0.016; *t*_(5992)_ = 2.29; *p* = 0.022; *p*_*SA*_ = 0.106; Supplementary Table S1). Furthermore, we correlated the random-effect coefficients (from the original and the surrogate maps) to both βPC and βpupil and observed a significant positive correlation between the participation coefficient and both pupils (Pearson’s *r* = 0.54, *p* = 0.02, *p*_*SA*_ = 0.036; Figure 4B) and mean drift rate (Pearson’s *r* = 0.70, *p* = 0.001, *p*_*SA*_ = 0.001; Figure 4C). This result shows the manner in which pupil diameter linearly shapes βBOLD cortical map through the engagement of the α2a receptor expression map. Importantly, although the fixed effect of α2a on βBOLD didn’t survive the spatial autocorrelation correction, the linear correlation of this effect with both βpupil and drift rate (between subjects) did survive the correction.

To further analyze the between-subject differences in the role of α2a receptor atlas in shaping the βBOLD map, we ran a separate linear model within each subject with α2a as a regressor and βBOLD of each region as the dependent variable (while also correcting for spatial autocorrelation using 5,000 surrogate maps). As can be seen in Figure 4B–C, we observed a dependency between the *p*_*SA*_ value, βpupil, and drift rate, in which the respective within-subject effects that survived the spatial autocorrelation correction are shown (*p*_*SA*_ < 0.05; marked circles in Figure 4B–C). Despite these results, there was no significant effect of α2a on βPC (Equation 9; β_α2a_ = 0.001 ± 0.003; *t*_(5992)_ = −0.51; *p* = 0.6), and no significant Pearson’s correlations were found between the random effects and both βpupil or drift rate (*r* = −0.24, *p* = 0.33; and *r* = −0.23, *p* = 0.341, respectively). However, we did find a significant effect of βBOLD on βPC (Equation 10; β = 0.0259 ± 0.006; *t*_(5992)_ = 3.96; *p* = 7.55 × 10^−5^; Supplementary Table S1 and Figure 4D). Together these results propose a closer link between pupil diameter, ascending neuromodulation, and the cortical neuromodulation dependent on α2a receptor density.

Finally, we observed a differential relationship between βPC and βBOLD depending on the large-scale module to which the regions were assigned. We expanded the former result by measuring, within each subject, the Pearson correlation between the βBOLD and βPC separately in each large-scale module (M1 being the modules assigned as red and blue, and M2 assigned as yellow and green; Figure 2). The results demonstrated a significant difference between modules, meaning that M1 has a higher correlation with βPC, in comparison with M2 (*t*_(17)_ = −12.99, *p* = 2.93 × 10^−10^, Supplementary Figure S5C). These results provided evidence that the adrenergic receptor distribution of α2a shapes the βBOLD activation map in proportion to the subject’s pupil diameter. Additionally, βBOLD activation map modulates (i.e., was related to) mesoscale integration, and mesoscale integration is related to pupil diameter. Based on these results, we hypothesize that the adrenergic system shapes the BOLD activity, which in turns shapes the topology of the network towards integration. However, future work is required in order to test this hypothesis more directly, for instance by combining optogenetic approaches with neuronal recordings in awake animals.

## DISCUSSION

Here, we leveraged a unique dataset to simultaneously track pupil diameter and network topology during an attentional demanding task with increasing attentional load. Our results provide integrative evidence that links the ascending arousal system to the mesoscale topological signature of the functional brain network during the processing of an attentionally demanding cognitive task. Pupil diameter was tracked with attentional load (Figure 1A) and was related to the speed of information accumulation as estimated by a drift diffusion model (Figure 1B–C). Additionally, we observed concurrent pupil dilations and adaptive mesoscale parametric topological changes as a function of task demands (Figures 2 and 3). Finally, we found evidence that topological reconfiguration was dependent on the regional activity and the genetic expression of the adrenergic receptors in the brain (Figure 4). Together, these results provide evidence for the manner in which the ascending arousal noradrenergic system reconfigures brain network topology so as to promote attentional performance according to task demands.

The relationship between performance and pupil diameter is consistent with the predictions of adaptive gain theory (Aston-Jones & Cohen, 2005). Within this framework, the locus coeruleus is proposed to adaptively alter its activity according to the demands imposed on the system. More specifically, the theory proposes that performance follows an inverted U-shaped relationship with arousal, such that maximal operational flexibility in the noradrenergic system is associated with optimal task performance (Arnsten et al., 2012; Robbins & Arnsten, 2009). We observed that load-related increases in pupil diameter, presumably due to increased activity in the ascending arousal system (Aston-Jones & Cohen, 2005; S. Joshi et al., 2016; Liu et al., 2017), relates closely with the activity and topology of the broader brain network (Figure 2), in a manner that is reflective of effective task performance (Figure 3). Similar effects have been described in animal models after a chemogenetic activation of the locus coeruleus, which strongly alters the large-scale network structure towards large-scale integration, specifically in regions with heightened adrenergic receptor expression (Zerbi et al., 2019). How these changes, which are likely related to the modulation of the neural gain that mediates effective connections between distributed regions of the brain (Shine et al., 2021; Shine et al., 2018), are traded off against requirements for specificity and flexibility remains an important open question for future research.

The addition of attentional load was found to alter the integration of mesoscale submodules, but not the higher level modular organization. This topological result is somewhat more targeted than those described in previous work (Shine, Breakspear, et al., 2019; Shine et al., 2016). While these differences may be related to disparities in the way that the data were analyzed, the results of our study do demonstrate that alterations in the cerebral network topology at a relatively local (i.e., submodular) level are crucial for effective task performance (Akiki & Abdallah, 2019). Additionally, our results replicate and expand upon a previous study (Mohr et al., 2016), in which the authors found that short-term practice on an attentional task was related to increased coupling between attentional networks and segregation among task-negative (DMN) and frontoparietal network (FPN). Our study replicates the graph theoretical results of that study, while also directly relating the findings to the architecture of the ascending neuromodulatory system. One potential explanation for these results comes from animal studies, in which rapid changes in pupil diameter have been compared with changes in neural population activity at the microscale (S. Joshi et al., 2016; McGinley et al., 2015; Reimer et al., 2014). These studies suggest that the ascending arousal system may be able to alter the topology of the network in a hierarchical manner that is commensurate with the spatiotemporal scale of the arousal systems’ capacity (Shine et al., 2016). Future work that integrates results across spatiotemporal scales is required to appropriately adjudicate the implications of this hypothesis.

Importantly, our approach is not without limitations. For one, the participation measures used in our linear mixed model were estimated at the mesoscale level, and hence derived from different modular partitions. Furthermore, the specificity of the pupillary response as a correlate of locus coeruleus (LC) activity is currently under active debate. For instance, in addition to the strong empirical links between the noradrenergic system and pupil dilation, there is also evidence that the pupil is dilated in concert with activity in the basal forebrain cholinergic system (Reimer et al., 2016), however it bears mention that both peripheral (Kaymak et al., 2018) and central cholinergic tone (Yüzgeç et al., 2018) are associated with pupillary constriction. There are more plausible physiological routes for the serotonergic system to dilate the pupil (via the excitation of the intermediolateral cell column), and in keeping with this, there is evidence that the serotonergic system is linked with pupil dilation (Cazettes et al., 2020). Nevertheless, it is important to take into account that the neuromodulatory arousal system is replete with complex interconnections (Avery & Krichmar, 2017; Briand et al., 2007; A. Joshi et al., 2017; Smiley et al., 1999). In addition, based on the current lack of a specific mechanism involving pupillary changes through the cholinergic system, it is highly probable that those correlations are due to indirect modulation of pupillary responses (e.g., via indirect neuromodulation mediated by the LC system). On the other hand, we acknowledge the limitations of the atlas receptor analysis and the linear model used in our study. More specific neurobiological properties of the receptor distributions are needed to make better inferences, and hence provide more accurate answers of their role in brain dynamics. For instance, it would be ideal to compare receptor distributions that incorporated layer-specific expression, as there are well-known cellular and circuit differences across layers in the cerebral cortex (Douglas & Martin, 2004; Palomero-Gallagher & Zilles, 2019). Importantly, taking into consideration the strong correlation between different genetic expression maps (Fornito et al., 2019), it is possible that the current correlation between ADRA2A expression and brain activity is a false positive caused by another neuroanatomical gradient strongly correlated to the ADRA2A. Therefore, future work studying the interaction between genetic expression of the neuromodulatory receptors, pupil diameter, and brain activity is needed. In spite of this limitation, we believe in the importance of integrating pupil diameter and receptor distribution in the analysis as the relationships between noradrenergic tone, brain activity, and network topology will help us to disentangle the mechanistic steps connecting the locus coeruleus system to both pupil diameter and brain dynamics.

In summary, we provide evidence linking mesoscale topological network integration, hierarchical organization, and BOLD dynamics in the human brain that increases in attentional load, thus providing further mechanistic clarity over the processes that underpin the adaptive gain model of noradrenergic function in the central nervous system.

## METHODS

### Participants

Eighteen right-handed individuals (age 19–26 years; five male) were included in this study. Exclusion criteria included standard contraindications for MRI; neurological disorders; and mental disorders or drug abuse. All participants gave written informed consent before the experiment.

### Parametric Motion Tracking Task

Each trial of the task involved the same basic pattern (Figure 1A): The task begins with a display presenting the objects (i.e., blue colored disks); after a 2.5-s delay, a subset of the disks turn red for another 2.5 s; all of the disks then return to blue (2.5 s) before they start moving randomly inside the tracking area. The participants’ job is to track the “target” dots on the screen while visually fixating at the cross located at the center of the screen. After a tracking period of ∼11 s, one of the disks is highlighted in green (a “probe”) and the subject is then asked to respond, as quickly as possible, as to whether the green probe object was one of the original target objects. The number of objects that subjects were required to attend to across the tracking period varied across trials. There were five trial types: passive viewing (PV), in which no target is assigned; and four load conditions, in which two to five targets were assigned for tracking. We operationalized attentional load as the linear effect of increasing task difficulty (i.e., the number of targets to be tracked).

The experiment was conducted using a blocked design, in which each block included the following: instruction (1 s); fixation (0.3 s, present throughout the rest of trial); object presentation (all objects were blue; 2.5 s); target assignment (i.e., the targets changed color from blue to red; 2.5 s); object representation (objects back to the original blue color; 2.5 s); object movement/attentional tracking (moving blue dots; 11 s); object movement cessation (0.5 s); and a final probe (color change to green and response; 2.5 s). The total duration of each trial was 22.8 s. Each condition was repeated four times in one fMRI run, which also included four separate fixation periods of 11 s each between five consecutive trials. All participants completed four separate runs of the experiment, each of which comprised 267 volumes. The order of the conditions was pseudorandom, such that the different conditions were grouped in sub-runs of triplets: PV, pseudorandom blocks of Loads 2 through 5, and a fixation trial. All objects were identical during the tracking interval and standard object colors were isoluminant (to minimize incidental pupillary responses during the task).

### Behavior and EZ-Diffusion Model

The EZ-diffusion model was used to interpret the performance measures from the task (Ratcliff & Rouder, 1998; Wagenmakers et al., 2007). This model considers the mean RT of correct trials, the standard deviation of the reaction time (SD-RT) across correct trials, and mean accuracy across the task, and computes from these a value for drift rate (*v*, Equation 1), boundary separation (*a*, Equation 2) , and non-decision time (Equation 3)—the three main parameters for the drift diffusion model (Ratcliff et al., 2016; Ratcliff & Rouder, 1998).

$$v=\text{sign}\left(P-\frac{1}{2}\right)\cdot 0.1\cdot \sqrt[{4}]{\frac{\log\left(\frac{P}{1-P}\right)\cdot \left[P^{2}\cdot \log\left(\frac{P}{1-P}\right)-P\cdot \log\left(\frac{P}{1-P}\right)+P-\frac{1}{2}\right]}{VRT},}$$ (1)

$$a=0.01\cdot \frac{\log\left(\frac{P}{1-P}\right)}{v},$$ (2)

$$Ter=MRT-\frac{a}{2\times v}\times \frac{\left(1-e^{-100\cdot v\cdot a}\right)}{\left(1+e^{-100\cdot v\cdot a}\right)},$$ (3)

in which *P* is the average performance (range between 0 and 1); sign is an operator that will be −1 if *P* < 0.5 or +1 if *P* > 0.5; *VRT* is the standard deviation of reaction time (in seconds); and *MRT* is the mean reaction time (in seconds).

### Pupillometry

Fluctuations in pupil diameter of the left eye were collected using an MR-compatible coil-mounted infrared EyeTracking system (NNL EyeTracking camera, NordicNeuroLab, Bergen, Norway), at a sampling rate of 60 Hz and recorded using the iView X Software (SensoMotoric Instruments, SMI GmbH, Germany). Blinks, artifacts, and outliers were removed and linearly interpolated (Wainstein et al., 2017). High-frequency noise was smoothed using a second-order 2.5-Hz low-pass Butterworth filter. To obtain the pupil diameter average profile for each level of attentional load (Figure 1B), data from each participant were normalized across each task block (corresponding to the five consecutive trials between fixations). This allowed us to correct for low-frequency baseline changes without eliminating the load effect and baseline differences due to load manipulations (Campos-Arteaga et al., 2020; Rojas-Líbano et al., 2019). Following this, a linear regression was performed in each time point using the task load as regressor and resulting in a “load effect” time series for each subject.

### MRI Data

Imaging data were collected on a Philips Achieva 3 Tesla MR-scanner, equipped with an eight-channel Philips SENSE head coil (Philips Medical Systems, Best, Netherlands) at the Intervention Centre, Oslo University Hospital, Norway. Functional data were collected using a BOLD-sensitive T2*-weighted echo-planar imaging sequence (36 slices, no gap; repetition time (TR), 2,2 s; echo time (TE), 30 ms; flip angle, 80°; voxel size, 3 × 3 × 3; field of view (FOV), 240 × 240 mm; interleaved acquisition). Anatomical T1-weighted images consisting of 180 sagittal-oriented slices were obtained using a turbo field echo pulse sequence (TR, 6.7 ms; TE, 3.1 ms; flip angle 8°; voxel size 1 × 1.2 × 1.2 mm; FOV, 256 × 256 mm).

### fMRI Data Preprocessing

After realignment (using FSL’s MCFLIRT), we used FEAT to unwarp the EPI images in the y-direction with a 10% signal loss threshold and an effective echo spacing of 0.333. Following noise-cleaning with FIX (custom training set for scanner, threshold 20, included regression of estimated motion parameters), the unwarped EPI images were then smoothed at 6-mm FWHM, and nonlinearly coregistered with the anatomical T1 to 2-mm isotropic MNI space. Temporal artifacts were identified in each dataset by calculating framewise displacement (FD) from the derivatives of the six rigid-body realignment parameters estimated during standard volume realignment (Power et al., 2014), as well as the root mean square change in BOLD signal from volume to volume (DVARS). Frames associated with FD > 0.25 mm or DVARS > 2.5% were identified; however, as no participants were identified with greater than 10% of the resting time points exceeding these values, no trials were excluded from further analysis. There were no differences in head motion parameters between the four sessions (*p* > 0.500). Following artifact detection, nuisance covariates associated with the six linear head movement parameters (and their temporal derivatives), DVARS, physiological regressors (created using the RETROICOR method), and anatomical masks from the cerebrospinal fluid and deep cerebral white matter were regressed from the data using the CompCor strategy (Behzadi et al., 2007). Finally, in keeping with previous time-resolved connectivity experiments (Gu et al., 2015), a temporal band pass filter (0.0071 < f < 0.125 Hz) was applied to the data.

### Brain Parcellation

Following preprocessing, the mean time series was extracted from 375 predefined regions of interest (ROIs). To ensure whole-brain coverage, we extracted the following: (a) 333 cortical parcels (161 and 162 regions from the left and right hemispheres, respectively) using the Gordon atlas (Gordon et al., 2016); (b) 14 subcortical regions from the Harvard-Oxford subcortical atlas (bilateral thalamus, caudate, putamen, ventral striatum, globus pallidus, amygdala, and hippocampus; https://fsl.fmrib.ox.ac.uk/); and (c) 28 cerebellar regions from the SUIT atlas (Diedrichsen et al., 2009) for each participant in the study.

### Time-Resolved Functional Connectivity and Network Analysis

To estimate functional connectivity between the 375 ROIs, we used the jackknife correlation (JC) approach (Thompson et al., 2018). Briefly, this approach estimates the static correlations between each pair of regions, and then recalculates the correlation between each pair after systematically removing each temporal “slice” of data (i.e., each TR). By subtracting the jackknifed correlation matrix from the original “static” matrix, the difference in connectivity at each slice from the static connectivity value can be used as an estimate of time-resolved functional connectivity between each pair of regions at each TR in a way that does not require windowing.

### Community Structure

The Louvain modularity algorithm from the Brain Connectivity Toolbox (Rubinov & Sporns, 2010) was used in combination with the JC to estimate both time-averaged and time-resolved community structure. The Louvain algorithm iteratively maximizes the modularity statistic, *Q*, for different community assignments until the maximum possible score of *Q* has been obtained (Equation 4).

$$Q_{T}=\frac{1}{v^{+}}\sum_{ij}\left(w_{ij}^{+}-e_{ij}^{+}\right)\delta_{M_{i}M_{j}}-\frac{1}{v^{+}+v^{-}}\sum_{ij}\left(w_{ij}^{-}-e_{ij}^{-}\right)\delta_{M_{i}M_{j}}.$$ (4)

**Equation 4**: Louvain modularity algorithm, where *v* is the total weight of the network (sum of all negative and positive connections); *w*_*ij*_ is the weighted and signed connection between regions *i* and *j*; *e*_*ij*_ is the strength of a connection divided by the total weight of the network; and *δ*_*M*_i_*M*_*j*__ is set to 1 when regions are in the same community and 0 otherwise. The + and − superscripts denote all positive and negative connections, respectively.

For each subject, we calculated the mean adjacency matrix from 1 TR before tracking until the end of the tracking period. Afterwards, a consensus partition was estimated across subjects. Finally, to identify multilevel structure in our data, we repeated the modularity analysis for each of the modules identified in the first step (Meunier et al., 2010; Meunier et al., 2009). With this final module assignment, we were afforded an estimate of the time-resolved, multilevel modularity (Q_*T*_) within each temporal window for each participant in the study.

### Regional Integration

Based on the group consensus community assignments, we estimated between-module connectivity using the participation coefficient, B_*T*_, which quantifies the extent to which a region connects across all modules (i.e., between-module strength; Equation 5). In our experiment, we used two separate community assignments, one for each of the modularity levels. In this manner we measure (a) how the first hierarchical-level (i.e., large-scale) topology changed during tracking across the complete brain; and (b) how the topology of the submodules changed across the task. These values were calculated in each time point using the time-resolved adjacency matrix across each load condition.

$$B_{iT}=1-\sum_{s=1}^{n_{M}}{\left(\frac{\kappa_{isT}}{\kappa_{iT}}\right)}^{2}.$$ (5)

**Equation 5**: Participation coefficient B_*iT*_, where κ_isT_ is the strength of the positive connections of region *i* to regions in module *s* at time *T*, and κ_iT_ is the sum of strengths of all positive connections of region *i* at time *T*. The participation coefficient of a region is therefore close to 1 if its connections are uniformly distributed among all the modules and 0 if all of its links are within its own module.

### Neurotransmitter Receptor Mapping

To investigate the potential correlates of mesoscale integration, we interrogated the neurotransmitter receptor signature of each region of the brain. We used the Allen Brain Atlas microarray atlas dataset (https://human.brain-map.org/; Hawrylycz et al., 2012) to identify the regional signature of genetic expression of the α2a subtype of the adrenergic receptor (ADRA2A). This receptor has been a priori related to cognitive function and attention (Arnsten & Haven, 2013), and is one of the most abundant adrenergic subtypes expressed in the cerebral cortex (Perez, 2020). This atlas contains postmortem samples of six donors that underwent microarray transcriptional characterization. The spatial map of α2a mRNA expression was obtained in volumetric 2-mm isotropic MNI space, following improved nonlinear registration and whole-brain prediction using variogram modeling (Gryglewski et al., 2018). We used these data instead of the native sample-wise values in the AHBA database to prevent bias that could occur because of spatial inhomogeneity of the sampled locations. We projected the volumetric α2a expression data onto the Gordon atlas with linear interpolation and calculated the mean value within each parcel using custom MATLAB codes.

## STATISTICAL ANALYSIS

### The Relationship Between Sympathetic Tone and Attentional Processing

We analyzed the between-subject effect of load on the behavioral, pupillometric, and fMRI-related variables by performing a two-level linear model analysis. In the first level, we used attentional load as a regressor (2 to 5) and—in independent models—the mean accuracy, reaction time, standard deviation of reaction time, drift rate, boundary criteria, and non-decision time as dependent variables (i.e., four values per subject). From this, we ran a two-tailed *t* test on the statistical effects (i.e., the β value from the regression, one for each subject; *N* = 18). Similarly, to calculate the load effect on pupil diameter, we calculated the average pupil diameter on each load condition within each subject. Then, we performed a first-level analysis in which we ran a linear regression in each time frame (1600 frames in total, corresponding to 26.6 seconds). This procedure resulted in one β timeseries (i.e., the statistical load effect on pupil diameter) for each subject across the trial (Supplementary Figure S1A). After this, we performed a right tailed *t* test in each frame across subjects (*n* = 18 in each frame) to find the periods of time where the β values where higher than 0. Finally, we corrected by false discovery rate (FDR; Benjamini & Yekutieli, 2001) for multiple testing, which resulted in a period of time in which the load effect was higher than 0 (light gray area in Figure 1A). The mean β values during this section was calculated in each subject and defined as “βpupil.” Finally, following the same pipeline, we calculated the effect of attentional load on the brain-related signals (i.e., BOLD, participation coefficient [PC], and modularity [Q]). The effect of load on BOLD was calculated running a separate linear model in each subject and region within each TR (18 subjects; 375 regions; 10 TRs; 4 load conditions), resulting in a matrix of β values of 18 × 375 × 10.

To evaluate the statistical effect of pupil diameter on accuracy, we performed a logistic linear mixed-effects model. We used the mean pupil diameter of the significant time period (Figure 1A) of the high load trials (Loads 4 and 5) and the accuracy (i.e., correct or incorrect) as the predictor variable of each trial, grouping by subject as the random effect. The statistical model is described in the following equation:

$$\text{Accuracy}∼\text{Pupil}+1+\left(\text{Pupil}+1|\text{Subject}\right).$$ (6)

### Network Integration Increases as a Function of Attentional Load

To evaluate whether the modularity of the network we observed was higher than chance, we generated 100 random networks in each hierarchical level (300 random networks in total), with a preserved degree distribution (using the MATLAB *randmio_und_signed* function from the Brain Connectivity Toolbox; Rubinov & Sporns, 2010). We calculated the modularity value of each random network and used the resultant values to populate a null distribution (Figure 2D).

We analyzed the statistical effect of pupil diameter on the participation coefficient both within and between subjects by performing a linear mixed model using the time-varying PC of the red submodule (Figure 3A) of each load as a dependent variable (*N* = 72), and the respective pupil diameter as a regressor, with grouping by subject. The statistical model is described in the following equation:

$$\text{PC}∼\text{Pupil}+1+\left(\text{Pupil}+1|\text{Subject}\right).$$ (7)

### Network Mesoscale Integration and Adrenergic Receptor Density

Expression of brain genetic atlas varies smoothly across the surface and thus is associated with nontrivial spatial autocorrelation that in turn violates the assumption of independence between samples (Burt et al., 2020; Markello & Misic, 2021; Vos de Wael et al., 2020). To account for the spatial autocorrelation in these brain maps, we used spatial autocorrelation null maps as implemented in Brain Surrogate Maps with Autocorrelated Spatial Heterogeneity (BrainSMASH) Python toolbox (Burt et al., 2020). A geodesic distance matrix of the atlas parcels using the surface of the Gordon atlas was obtained to build the surrogates using BrainSMASH functions. We generated 5,000 null maps that were used to generate null distribution of the different statistics corrected by spatial autocorrelation.

We measure the statistical difference in the receptor density between submodules by a two-tailed *t* test between each pair of modules. The same procedure was performed using the surrogate maps to generate a null distribution of *t* statistics. To evaluate the effect of the density of each adrenergic receptor on the neural activity in the attentional task, we built a linear mixed model aimed at predicting regional differences in BOLD activity and participation coefficient. We created a model using the receptor density atlas of α2a receptor to predict parametric BOLD activity (i.e., linear increase of BOLD activity with task load) during tracking (Equation 8). To evaluate the relationship between BOLD activity, adrenergic receptor expression, and changes in participation coefficient as a function of attentional load, we tested two models: one using the adrenergic receptor density as independent factor (Equation 9), and another using the parametric BOLD effect as an independent factor (Equation 10). Additionally, we assessed the across-subject variability using the subjects’ ID as a grouping variable in order to evaluate the random effects on the independent factor. We corrected the spatial autocorrelation by running the same model using 5,000 surrogate maps. Then we used the fixed-effect null distribution to calculate the *p*_SA_ (i.e., the probability of finding the fixed effect within the 95th percentile of the null distribution). The deterministic part of the model is expressed in the following equations (Wilkinson & Rogers, 1973):

$$\text{βBOLD}∼\alpha 2a+1+\left(\alpha 2a+1|\text{Subject}\right),$$ (8)

$$\text{βPC}∼\alpha 2a+1+\left(\alpha 2a+1|\text{Subject}\right),$$ (9)

$$\text{βPC}∼\text{BOLD}+1+\left(\text{BOLD}+1|\text{Subject}\right),$$ (10)

where *PC* is the parametric effect of mesoscale participation coefficient (i.e., βPC), BOLD is the parametric effect of load on BOLD activity during tracking for each region, and α2a are the regional densities of the respective adrenergic receptor atlas. We then correlated the random-effects parameters to pupil diameter responses and behavior and then compared these with the Pearson’s correlation of the null distribution using the random effect of the surrogate maps. Finally, we performed a linear model within each subject with α2a as a regressor and βBOLD as dependent variable. Again, the statistical effect (i.e., β value) was compared against the null distribution when performing the regression using the surrogate maps (Figure 4B–C).

## ACKNOWLEDGMENTS

We thank P. Billeke for his thoughtful comments on our manuscript.

## DATA AND CODE AVAILABILITY

The anonymized preprocessed fMRI and pupillometry data can be found at https://figshare.com/articles/dataset/MOT_data_mat/13244504 (Wainstein et al., 2020). The ADRA2A expression atlas can be downloaded from https://www.meduniwien.ac.at/neuroimaging/mRNA.html. All analysis of the fMRI and pupil diameter data were performed on MATLAB 2020a. The surrogate maps of the ADRA2A atlas were generated on Python. Documented code for reproducing the analyses is provided in https://github.com/gabwainstein/MOT (Wainstein, 2021). Supporting information for this article is available at https://doi.org/10.1162/netn_a_00205.

## AUTHOR CONTRIBUTIONS

Gabriel Wainstein: Conceptualization; Formal analysis; Investigation; Methodology; Visualization; Writing – original draft; Writing – review & editing. Daniel Rojas-Líbano: Visualization; Writing – original draft; Writing – review & editing. Vicente Medel: Formal analysis; Writing – review & editing. Dag Alnæs: Conceptualization; Data curation; Funding acquisition; Investigation; Methodology; Writing – review & editing. Knut K. Kolskår: Conceptualization; Investigation; Validation; Writing – review & editing. Tor Endestad: Conceptualization; Investigation; Methodology; Writing – review & editing. Bruno Laeng: Conceptualization; Investigation; Writing – review & editing. Tomas Ossandon: Supervision; Writing – review & editing. Nicolás Crossley: Validation; Writing – review & editing. Elie Matar: Conceptualization; Writing – review & editing. James M. Shine: Conceptualization; Data curation; Formal analysis; Investigation; Methodology; Project administration; Resources; Supervision; Validation; Writing – original draft; Writing – review & editing.

## FUNDING INFORMATION

James M. Shine, the University of Sydney Robinson Fellowship. James M. Shine, National Health and Medical Research Council (https://dx.doi.org/10.13039/501100000925), Award ID: GNT1156536. Gabriel Wainstein, Becas Chile.

## TECHNICAL TERMS

Network: Theoretical representation of a system. Each part is represented as a node and the connection between nodes as edges.
Integration: Number of connections of a region or set of regions outside its own module.
Locus coeruleus: Principal noradrenergic neuromodulatory nuclei located in the brain stem. Projects towards the central nervous system.
Noradrenaline: A monoaminergic neurotransmitter that modulates the neuronal activity of the target populations in the nervous system.
Drift rate: A marker of the speed of the accumulation of decision evidence during the decision-making process.
Module: Group of nodes that have more connection strength between them than to the rest of the system.
Modularity: Quality function that represents the amount of connection of nodes within each module to nodes in the same module.
Load effect: Statistical linear effect of the task load (i.e., difficulty) on a given dependent variable.
α2a: Noradrenergic G-protein coupled receptor. It is the main adrenergic presynaptic and postsynaptic receptor subtype in the human brain.
ADRA2A atlas: RNA expression atlas of the α2a. It is used as an indirect marker of the density of the α2a receptor.

## Supporting Information

https://github.com/gabwainstein/MOT

https://doi.org/10.6084/m9.figshare.13244504.v1
