# Supplementary material for: The ascending arousal system promotes optimal performance through mesoscale network integration in a visuospatial attentional task
Source: Netw Neurosci. 2021 Nov 30;5(4):890–910. doi: 10.1162/netn_a_00205 (PMC8746119; doi:10.1162/netn_a_00205)
Supplement: Supplementary file 1 [file netn-05-890-s001.pdf]

Supplementary Material for

The ascending neuromodulatory system promotes optimal performance through meso-scale network integration in a visuospatial attentional task

Authors

Gabriel Wainstein[1], Daniel Rojas-Libano[2], Vicente Medel[1,9], Dag Alnæs[3,4], Knut K. Kolskår[3, 5,6], Tor Endestad[5,7,8], Bruno Laeng[5,7], Tomas Ossandon[9,10], Nicolás Crossley[9], Elie Matar[1] and James M. Shine[1,11*]

Affiliations

1 Brain and Mind Centre, The University of Sydney, Sydney, NSW, Australia

2 Centro de Neurociencia Humana y Neuropsicología, Facultad de Psicología, Universidad Diego Portales, Santiago, Chile

3 NORMENT, Division of Mental Health and Addiction, University of Oslo, and Oslo University Hospital, Oslo, Norway

4 Bjørknes University College, Oslo, Norway

5 Department of Psychology, University of Oslo, Oslo, Norway

6 Sunnaas Rehabilitation Hospital HT, Nesodden, Norway

7 RITMO Centre for Interdisciplinary Studies in Rhythm, Time and Motion, University of Oslo, Norway

8 Helgelandssykehuset Mosjøen, Helse Nord, Norway

9 Department of Psychiatry, School of Medicine, Pontificia Universidad Católica de Chile, Santiago, Chile

10 Institute for Biological and Medical Engineering, Schools of Engineering, Medicine and Biological Sciences, Pontificia Universidad Católica de Chile, Chile

11 Centre for Complexity, The University of Sydney, Sydney, NSW, Australia

Corresponding author

* James M. Shine – mac.shine@sydney.edu.au

Supplementary Figures

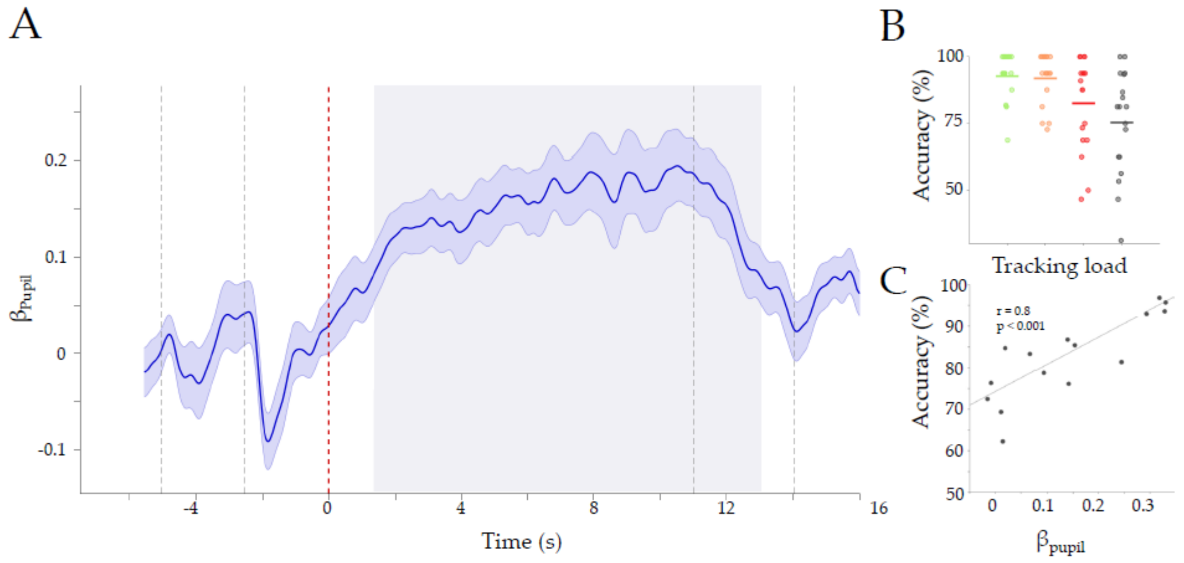

Supplementary Figure S1: A) regression parameter of the pupil diameter (β_{Pupil}) – grey area represents the significant time period where the group $\beta_{Pupil} > 0$ (FDR corrected, $p_{FDR} < 0.01$); B) Accuracy across loads, each dot is the group average. Colors represent Load 2 to 5 in green, orange, red and black, respectively; C) Pearson correlation between the average β_{Pupil} during the significant period (grey area in A), to the average Accuracy. There is a significant positive correlation (Pearson r = 0.8, $p = 7.0 \times 10^{-5}$).

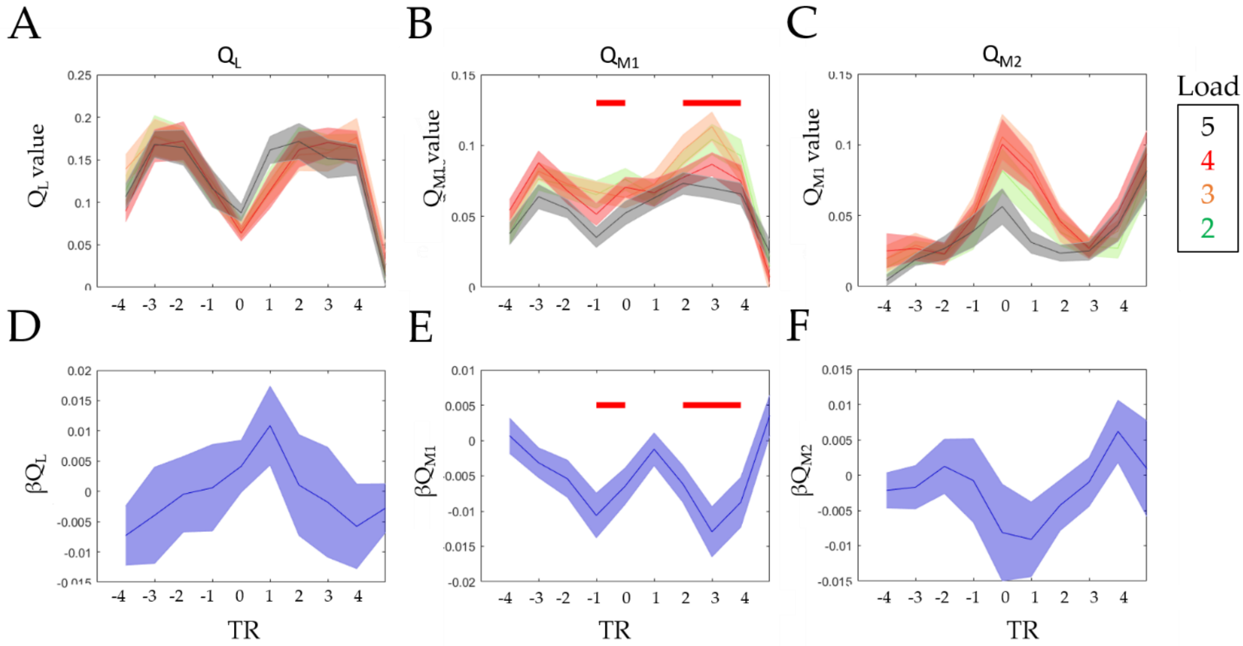

Supplementary Figure S2: Modularity time series for each attentional load and the parametric change across loads. In red (B and E) we show the significant time windows ($p_{FDR} < 0.05$, $\beta \neq 0$), which were only found for the Q_{M1} sub module (module red/blue in the main text). x-axis is Repetition Time (TR) from tracking onset (TR = 0). Lines represent the group average, and the shaded area is the standard error of the mean. Colors in A-C represent Load 2 to 5 in green, orange, red and black, respectively.

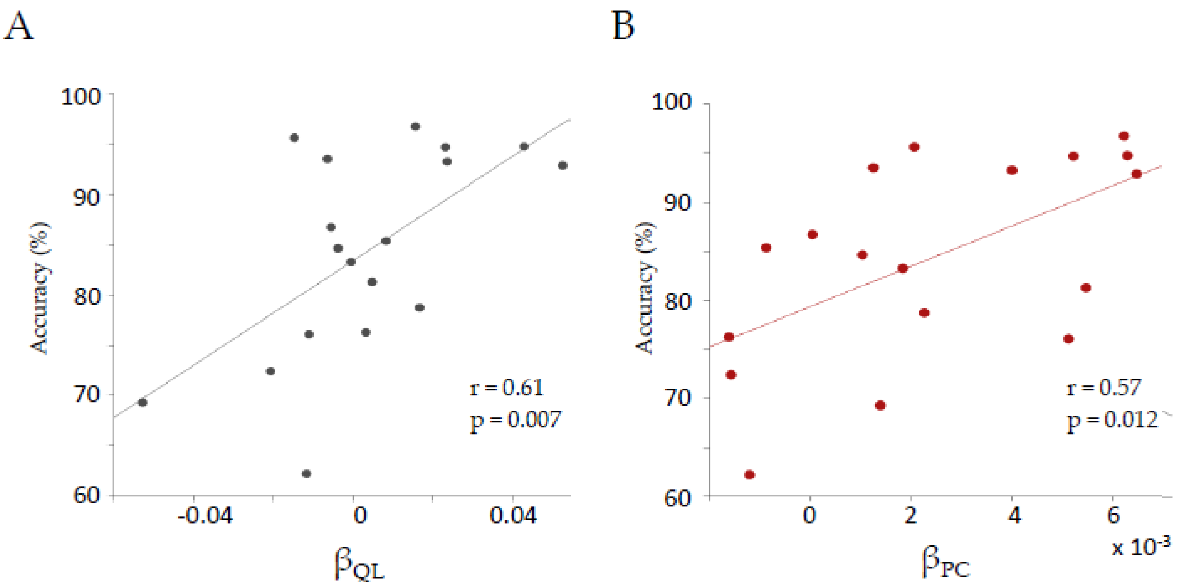

Supplementary Figure S3: Topological parametric effect (β_{QL} and β_{PC}) correlation to Accuracy (A; Pearson r = 0.61, P = 0.007) and (B; Pearson r = 0.57, P = 0.012).

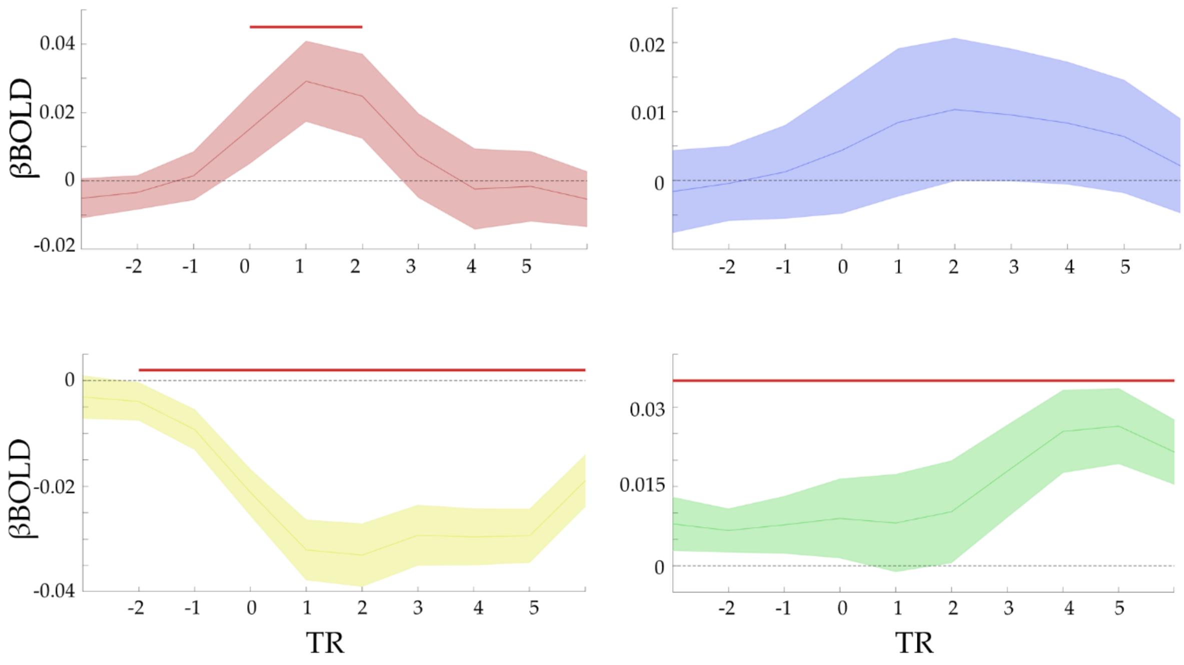

Supplementary Figure S4: Effect of attentional load on Bold activity. The statistical effect of load on bold is shown (βBOLD) for each TR (0 marks the start of tracking). Each plot represents the mean activity of each module. The colors represent the module assignment as in Figure 2 in main text. The red line in each plot shows the significant period of a two-tailed *t*-test ($\beta \neq 0$, $p_{FDR} < 0.05$) corrected by FDR.

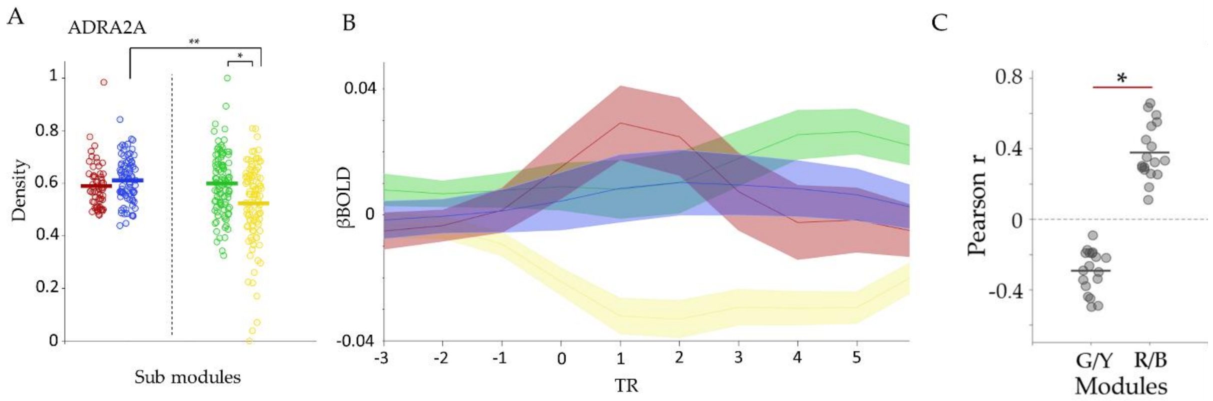

Supplementary Figure S5: A) Receptor density of α2a. The standardized density of the ADRA2A receptor expression, for each of the sub modules is shown. A two-tailed t-test was performed comparing the expression profiles within each pairs of sub-modules which was compared with 5000 surrogate maps accounting for spatial autocorrelation. The *p* values based on the null distribution is shown with significance levels * $p < 0.05$ ** $p < 0.01$. B) Bold load effect (βBOLD) time series during a trial within each sub-module. The shaded area corresponds to standard error across regions within the respective module. C) Each marker represent the Pearson correlation between β_{BOLD} and β_{PC} within each subject and separately for each large-scale module; significant differences are found between the two large scale module across the group ($t_{(17)} = -12.99$, $p = 2.93 \times 10^{-10}$). M1 is the red/blue, and M2 the yellow/green, as in Figure 2.

Supplementary Table S1: Mixed effect models relating α2a receptors atlas, Bold parametric effect, parametric participation coefficient (PC). Then regressors for both the fixed effect and the random effect (with subjects as grouping factor) is shown with the respective t test and p value. A significant effect is

Model 1	BOLD ~ α2a + 1 + (α2a + 1 | Subject)				
	**Fixed effect**			Random effect	
	Beta ± σ	t (5992)	*p* value	Beta	CI (95%)
β_0	-0.023 ± 0.01	-2.3753	0.017	0.034	-
$\beta_{\alpha 2a}$	0.037± 0.016	2.29	0.022	0.056	[0.053, 0.060]
Stats	AIC	BIC	LogLikelihood	Deviance	
	-11985	-11944	5998.3	-11997	

Model 2	PC ~ α2a + 1 + (α2a + 1 | Subject)				
	**Fixed effect**			Random effect	
	Beta ± σ	t (5992)	*p* value	Beta	CI (95%)
β_0	0.004 ± 0.002	1.871	0.061	0.009	[0.006, 0.013]
$\beta_{\alpha 2a}$	-0.001±0.003	-0.51	0.6	0.010186	[0.006, 0.017]
Stats	AIC	BIC	LogLikelihood	Deviance	
	-31198	-31158	15605	-31210	

Model 3	PC ~ BOLD + 1 + (BOLD + 1 | Subject)				
	**Fixed effect**			Random effect	
	Beta ± σ	t (5992)	*p* value	Beta	CI (95%)
β_0	0.003 ± 0.001	2.784	0.0053	0.005	[0.003, 0.007]
β_{BOLD}	0.0259 ± 0.006	3.961	7.55×10^{-5}	0.025	[0.017, 0.037]
Stats	AIC	BIC	LogLikelihood	Deviance	
	-31346	-31306	15679	-31358	

shown in the fixed effect of model 1 and model 3. Random effects were significant in the three models.

Supplementary Table S2: list of all 375 pre-defined regions-of-interest (ROI) and its module assignment. There are 333 cortical parcels (161 and 162 regions from the left and right hemispheres, respectively) using the Gordon atlas (4), 14 subcortical regions from Harvard-Oxford subcortical atlas (bilateral thalamus, caudate, putamen, ventral striatum, globus pallidus, amygdala and hippocampus; http://fsl.fmrib.ox.ac.uk/), and 28 cerebellar regions from the SUIT atlas (5). The table is grouped by module assignment (i.e. blue; red; green and yellow; Figure 2 of main text); the network they belong, and the hemisphere when possible to the left and right column, marked in L or R, respectively. The regions that where used in the circle plot from Figure 2B (main text) are shaded in dark grey. DAN, dorsal attention; VN, visual; FPN, frontoparietal; SN, salience; CO, cingulo-opercular; VAN, ventral attention; SMm, somatomotor mouth; SMh, somatomotor hand; RSpN, retrosplenial; FTPN, frontotemporal; DMN, default mode; AN, auditory; CPN, cinguloparietal; SubC, subcortex; Cer, Cerebellar.

Parcel ID	Hem	Network	Region	Parcel ID	Hem	Community	Region	Module
65	L	AN	L_PI	224	R	AN	R_PIns	Blue
67	L	AN	L_STG	233	R	AN	R_STG	Blue
69	L	AN	L_STG	181	R	CON	R_mPMA	Blue
104	L	AN	L_LPCG	185	R	CON	R_rACC	Blue
63	L	CON	L_OPJ	187	R	CON	R_rACC	Blue
76	L	CON	L_Mins	188	R	CON	R_rACC	Blue
82	L	CON	L_Sins	219	R	CON	R_OPJ	Blue
103	L	CON	L_OPJ	238	R	CON	R_Sins	Blue
105	L	CON	L_OPJ	245	R	CON	R_Mins	Blue
112	L	CON	L_LPCG	246	R	CON	R_Mins	Blue
93	L	CPN	L_mOPJ	274	R	CON	R_sAI	Blue
43	L	DAN	L_FEF	318	R	CON	R_FP	Blue
52	L	DAN	L_SPL	250	R	DAN	R_IFG	Blue
55	L	DAN	L_LSPL	220	R	DMN	R_OPJ	Blue
88	L	DAN	L_SPL	257	R	DMN	R_sPCC	Blue
92	L	DAN	L_mSPL	167	R	FPN	R_SPL	Blue
95	L	DAN	L_pOPJ	272	R	FPN	R_DLPFC	Blue
106	L	DAN	L_lPMA	280	R	None	R_mPFC	Blue
121	L	None	L_OFC	284	R	None	R_mPFC	Blue
122	L	None	L_OFC	285	R	None	R_mPFC	Blue
123	L	None	L_OFC	286	R	None	R_mPFC	Blue
124	L	None	L_OFC	287	R	None	R_mPFC	Blue
125	L	None	L_OFC	288	R	None	R_mPFC	Blue
35	L	SMh	L_mS1	302	R	None	R_vVis	Blue
37	L	SMh	L_mM1	303	R	None	R_vVis	Blue
57	L	SMh	L_S1	306	R	None	R_vVis	Blue
58	L	SMh	L_S1	195	R	SMh	R_mM1	Blue
3	L	SMm	L_lM1	164	R	SMm	R_lM1	Blue
39	L	SMm	L_lM1	197	R	SMm	R_lM1	Blue
59	L	SMm	L_S1	212	R	SMm	R_lM1	Blue
83	L	SN	L_Ains	247	R	SN	R_Ains	Blue
61	L	VAN	L_TOJ	221	R	VAN	R_TOJ	Blue
75	L	VAN	L_Sins	241	R	VAN	R_FO	Blue
79	L	VAN	L_FO	242	R	VAN	R_FO	Blue
158	L	VAN	L_PMA	256	R	VN	R_smVis	Blue
90	L	VN	L_smVis	258	R	VN	R_lVis	Blue

97	L	VN	L_lVis	263	R	VN	R_V1	Blue
98	L	VN	L_lVis	307	R	VN	R_V1	Blue
139	L	VN	L_lVis	308	R	VN	R_V1	Blue
141	L	VN	L_V1	309	R	VN	R_V1	Blue
**Subcortical and cerebellar regions**								
353		Cer	Vermis_VI	354		Cer	Right_VI	Blue
356		Cer	Vermis_CrusI	362		Cer	Vermis_VIIb	Blue
361		Cer	Left_VIIb	365		Cer	Vermis_VIII a	Blue
370		Cer	Left_IX	340		SubC	Left Accumbens	Blue
374		Cer	Vermis_X	342		SubC	Right Caudate	Blue
337		SubC	Left Pallidum	347		SubC	Right Accumbens	Blue
335		SubC	Left Caudate					Blue
64	L	AN	L_OPJ	230	R	AN	R_OPJ	Red
21	L	CON	L_PCC	180	R	CON	R_mPMA	Red
34	L	CON	L_mPMA	192	R	CON	R_mPMA	Red
40	L	CON	L_dPMA	196	R	CON	R_mPMA	Red
84	L	CON	L_sAIns	198	R	CON	R_dPMA	Red
41	L	DAN	L_FEF	223	R	CON	R_OPJ	Red
42	L	DAN	L_FEF	248	R	CON	R_sAIns	Red
49	L	DAN	L_dPMA	249	R	CON	R_sAIns	Red
51	L	DAN	L_SPL	189	R	DAN	R_mSPL	Red
87	L	DAN	L_SPL	199	R	DAN	R_FEF	Red
91	L	DAN	L_mSPL	203	R	DAN	R_FEF	Red
2	L	SMh	L_mS1	208	R	DAN	R_FEF	Red
30	L	SMh	L_mS1	211	R	DAN	R_SPL	Red
47	L	SMh	L_M1	163	R	SMh	R_mS1	Red
48	L	SMh	L_PMA	193	R	SMh	R_mS1	Red
50	L	SMh	L_S2	205	R	SMh	R_PMA	Red
54	L	SMh	L_lS1	206	R	SMh	R_PMA	Red
56	L	SMh	L_S2	207	R	SMh	R_PMA	Red
60	L	VAN	L_TOJ	209	R	SMh	R_S1	Red
5	L	VN	L_dVis	210	R	SMh	R_S1	Red
99	L	VN	L_lVis	213	R	SMh	R_lS1	Red
138	L	VN	L_iV1	214	R	SMh	R_S1	Red
				215	R	SMh	R_S1	Red
				217	R	SMh	R_S1	Red
				270	R	SMh	R_lS1	Red
				228	R	VAN	R_TOJ	Red
				166	R	VN	R_sVis	Red
				251	R	VN	R_SPL	Red
				255	R	VN	R_smVis	Red
				264	R	VN	R_lV1	Red
				267	R	VN	R_lVis	Red
				252	R	DAN	R_pSPL	Red
				262	R	DAN	R_SPL	Red
				271	R	DAN	R_dPMA	Red
				275	R	DAN	R_dPMA	Red
**Subcortical and cerebellar regions**								

364		Cer	Left_VIIIa	363		Cer	Right_VIIb	Red
367		Cer	Left_VIIIb	366		Cer	Right_VIIIa	Red
373		Cer	Left_X	369		Cer	Right_VIIIb	Red
359		Cer	Vermis_CrusII	375		Cer	Right_X	Red
				372		Cer	Right_IX	Red
68	L	AN	L_STG	183	R	SN	R_dACC	Green
22	L	CON	L_MCC	317	R	CON	R_SFG	Green
147	L	CON	L_FP	254	R	CPN	L_mOPJ	Green
153	L	CON	L_FP	236	R	DAN	R_IFG	Green
89	L	CPN	L_mOPJ	253	R	DAN	R_pSPL	Green
74	L	DAN	L_FO	266	R	DAN	R_ITG	Green
100	L	DAN	L_ITG	162	R	DMN	R_PCC	Green
107	L	DAN	L_lPMA	165	R	DMN	R_SFG	Green
110	L	DAN	L_IFG	200	R	DMN	R_SFG	Green
113	L	DAN	L_lPMA	225	R	DMN	R_ITG	Green
155	L	DAN	L_DLPFC	259	R	DMN	R_OPJ	Green
1	L	DMN	L_PCC	316	R	DMN	R_SFG	Green
4	L	DMN	L_SFG	325	R	DMN	R_SFG	Green
6	L	DMN	L_OPJ	326	R	DMN	R_SFG	Green
25	L	DMN	L_pSMA	168	R	FPN	R_DLPFC	Green
94	L	DMN	L_LOFJ	327	R	FPN	R_SFG	Green
114	L	DMN	L_FP	328	R	FPN	R_DLPFC	Green
126	L	DMN	L_STS	170	R	FPN	R_ITG	Green
154	L	DMN	L_SFG	182	R	FPN	R_pSMA	Green
156	L	DMN	L_SFG	240	R	FPN	R_IFG	Green
157	L	DMN	L_SFG	260	R	FPN	R_SPL	Green
7	L	FPN	L_IFG	261	R	FPN	R_SPL	Green
24	L	FPN	L_rACC	273	R	FPN	R_DLPFC	Green
78	L	FPN	L_FP	276	R	FPN	R_dPMA	Green
96	L	FPN	L_SPL	277	R	FPN	R_FP	Green
108	L	FPN	L_DLPFC	319	R	FPN	R_FP	Green
109	L	FPN	L_DLPFC	320	R	FPN	R_FP	Green
148	L	FPN	L_FP	281	R	None	R_mPFC	Green
149	L	FPN	L_FP	174	R	RTN	R_RSp	Green
115	L	None	L_FP	294	R	RTN	R_vmVis	Green
14	L	RTN	L_RSp	295	R	RTN	R_vmVis	Green
38	L	SMh	L_S1	313	R	RTN	R_HF	Green
45	L	SMh	L_M1	190	R	SMh	R_mS1	Green
46	L	SMh	L_M1	222	R	VAN	R_TOJ	Green
23	L	VAN	L_pSMA	229	R	VAN	R_TOJ	Green
62	L	VAN	L_STS	231	R	VAN	R_FO	Green
80	L	VAN	L_iAI	237	R	VAN	R_FO	Green
86	L	VAN	L_FO	333	R	VAN	R_STS	Green
8	L	VN	L_vmVis	169	R	VN	R_vmVis	Green
15	L	VN	L_V1	175	R	VN	R_V1	Green
16	L	VN	L_V1	176	R	VN	R_FFG	Green
17	L	VN	L_V1	177	R	VN	R_FFG	Green
20	L	VN	L_RSp	265	R	VN	R_lV1	Green
131	L	VN	L_vlVis	293	R	VN	R_vmVis	Green
132	L	VN	L_vlVis	298	R	VN	R_vVis	Green
136	L	VN	L_vlVis	299	R	VN	R_vVis	Green
137	L	VN	L_lV1	310	R	VN	R_V1	Green

140	L	VN	L_V1	311	R	VN	R_V1	Green
**Subcortical and cerebellar regions**								
348		Cer	Left_I_IV	357		Cer	Right_CrusI	Green
350		Cer	Left_V	351		Cer	Right_V	Green
355		Cer	Left_CrusI	360		Cer	Right_CrusII	Green
358		Cer	Left_CrusII					Green
352		Cer	Left_VI					Green
339		SubC	Left Amygdala					Green
10	L	AN	L_PI					Yellow
66	L	AN	L_STG	171	R	AN	R_PIns	Yellow
70	L	AN	L_PIns	227	R	AN	R_STG	Yellow
77	L	AN	L_PIns	232	R	AN	R_STG	Yellow
102	L	AN	L_LPCG	239	R	AN	R_PIns	Yellow
160	L	AN	L_STS	244	R	AN	R_PIns	Yellow
27	L	CON	L_rACC	268	R	AN	R_LPCG	Yellow
28	L	CON	L_rACC	269	R	AN	R_LPCG	Yellow
71	L	CON	L_MIns	329	R	AN	R_PIns	Yellow
72	L	CON	L_MIns	330	R	AN	R_STG	Yellow
81	L	CON	L_Sins	234	R	CON	R_Mins	Yellow
101	L	CON	L_LPCG	235	R	CON	R_Mins	Yellow
111	L	CON	L_LPCG	173	R	CPN	R_PCC	Yellow
12	L	CPN	L_PCC	184	R	DMN	R_mPFC	Yellow
26	L	DMN	L_PCC	186	R	DMN	R_PCC	Yellow
44	L	DMN	L_SFG	278	R	DMN	R_FP	Yellow
116	L	DMN	L_mPFC	279	R	DMN	R_mPFC	Yellow
117	L	DMN	L_mPFC	290	R	DMN	R_STG	Yellow
127	L	DMN	L_STS	315	R	DMN	R_SFG	Yellow
145	L	DMN	L_SFG	321	R	DMN	R_SFG	Yellow
146	L	DMN	L_SFG	322	R	DMN	R_SFG	Yellow
150	L	DMN	L_mSFG	323	R	DMN	R_SFG	Yellow
151	L	DMN	L_SFG	324	R	DMN	R_SFG	Yellow
152	L	DMN	L_dACC	331	R	DMN	R_STG	Yellow
9	L	FPN	L_ITG	226	R	VAN	R_STS	Yellow
85	L	VAN	L_FO	243	R	VAN	R_iAI	Yellow
161	L	VAN	L_STS	332	R	VAN	R_STS	Yellow
11	L	None	L_HF	172	R	None	R_TempP	Yellow
18	L	None	L_FFG	178	R	None	R_FFG	Yellow
19	L	None	L_PCC	179	R	None	R_PCC	Yellow
73	L	None	L_IFG	282	R	None	R_mPFC	Yellow
118	L	None	L_ITG	283	R	None	R_mPFC	Yellow
119	L	None	L_OFC	289	R	None	R_STG	Yellow
120	L	None	L_OFC	291	R	None	R_STG	Yellow
128	L	None	L_TempP	292	R	None	R_TempP	Yellow
129	L	None	L_TempP	296	R	None	R_vVis	Yellow
133	L	None	L_vlVis	297	R	None	R_vVis	Yellow
134	L	None	L_TempP	300	R	None	R_vVis	Yellow
135	L	None	L_TempP	301	R	None	R_vVis	Yellow
142	L	None	L_PHG	304	R	None	R_vVis	Yellow
144	L	None	L_PHG	305	R	None	R_vVis	Yellow
159	L	None	L_STG	312	R	None	R_HF	Yellow
13	L	RTN	L_RSp	314	R	None	R_mTemp	Yellow

130	L	RTN	L_vlVis	191	R	SMh	R_mM1	Yellow
143	L	RTN	L_PHG	194	R	SMh	R_mM1	Yellow
31	L	SMh	L_mS1	201	R	SMh	R_M1	Yellow
32	L	SMh	L_mM1	202	R	SMh	R_M1	Yellow
33	L	SMh	L_mM1	204	R	SMh	R_mM1	Yellow
36	L	SMh	L_mM1	216	R	SMh	R_S1	Yellow
53	L	SMm	L_lM1	218	R	SMm	R_lS1	
29	L	SN	L_dACC					Yellow
**Sub cortical and cerebellar regions**								
368		Cer	Vermis_VIIIb	349		Cer	Right_I_IV	Yellow
371		Cer	Vermis_IX	341		SubC	Right Thalamus	Yellow
334		SubC	Left Thalamus	343		SubC	Right Putamen	Yellow
336		SubC	Left Putamen	344		SubC	Right Pallidum	Yellow
338		SubC	Left Hippocampus	345		SubC	Right Hippocampus	Yellow
				346		SubC	Right Amygdala	Yellow
